# Transcriptional regulation and spatial interactions of head-to-head genes

**DOI:** 10.1186/1471-2164-15-519

**Published:** 2014-06-24

**Authors:** Yunqin Chen, Yixue Li, Jia Wei, Yuan-Yuan Li

**Affiliations:** Shanghai Center for Bioinformation Technology, Shanghai, P R China; AstraZeneca, R&D Information, 199 Liangjing Road, Building 2, Shanghai, 201203 China; Key Lab of Systems Biology, Shanghai Institutes for Biological Sciences, Chinese Academy of Sciences, Shanghai, P R China

## Abstract

**Background:**

In eukaryotic genomes, about 10% of genes are arranged in a head-to-head (H2H) orientation, and the distance between the transcription start sites of each gene pair is closer than 1 kb. Two genes in an H2H pair are prone to co-express and co-function. There have been many studies on bidirectional promoters. However, the mechanism by which H2H genes are regulated at the transcriptional level still needs further clarification, especially with regard to the co-regulation of H2H pairs. In this study, we first used the Hi-C data of chromatin linkages to identify spatially interacting H2H pairs, and then integrated ChIP-seq data to compare H2H gene pairs with and without evidence of spatial interactions in terms of their binding transcription factors (TFs). Using ChIP-seq and DNase-seq data, histones and DNase associated with H2H pairs were identified. Furthermore, we looked into the connections between H2H genes in a human co-expression network.

**Results:**

We found that i) Similar to the behaviour of two genes within an H2H pair (intra-H2H pair), a gene pair involving two distinct H2H pairs (inter-H2H pair) which interact with each other spatially, share common transcription factors (TFs); ii) TFs of intra- and inter-H2H pairs are distributed differently. Factors such as HEY1, GABP, Sin3Ak-20, POL2, E2F6, and c-MYC are essential for the bidirectional transcription of intra-H2H pairs; while factors like CTCF, BDP1, GATA2, RAD21, and POL3 play important roles in coherently regulating inter-H2H pairs; iii) H2H gene blocks are enriched with hypersensitive DNase and modified histones, which participate in active transcriptions; and iv) H2H genes tend to be highly connected compared with non-H2H genes in the human co-expression network.

**Conclusions:**

Our findings shed new light on the mechanism of the transcriptional regulation of H2H genes through their linear and spatial interactions. For intra-H2H gene pairs, transcription factors regulate their transcriptions through bidirectional promoters, whereas for inter-H2H gene pairs, transcription factors are likely to regulate their activities depending on the spatial interaction of H2H gene pairs. In this way, two distinctive groups of transcription factors mediate intra- and inter-H2H gene transcriptions respectively, resulting in a highly compact gene regulatory network.

**Electronic supplementary material:**

The online version of this article (doi:10.1186/1471-2164-15-519) contains supplementary material, which is available to authorized users.

## Background

**Figure 1:**
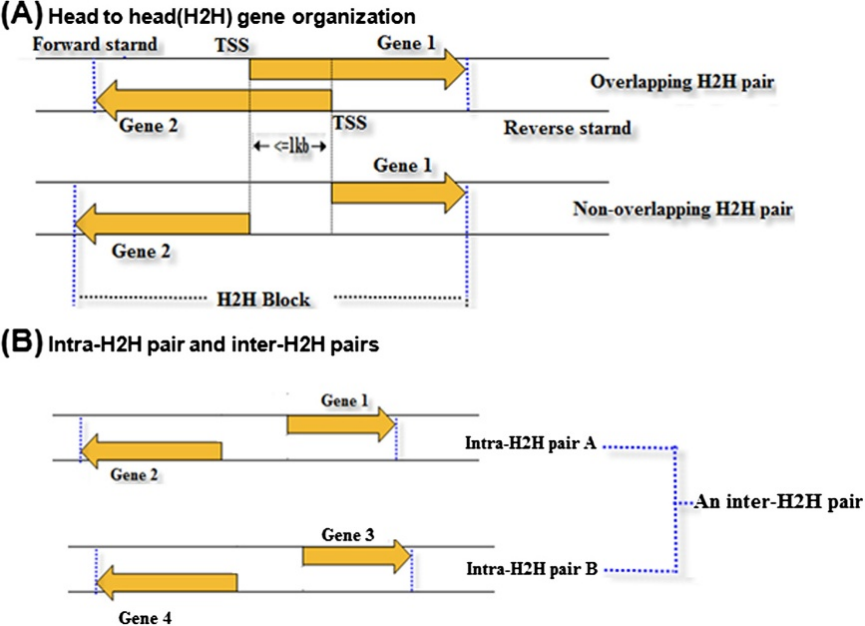
**The definition of head to head (H2H) gene organization and intra/inter-H2H pairs. (A)** Head to head (H2H) gene organization. The region between the two transcription start sites is called a bidirectional promoter and the region between the ends of two genes is an H2H block. **(B)** Intra-H2H pairs and an inter-H2H pair. An inter-H2H pair involves two intra-H2H gene pairs.

More than 10% of human adjacent protein-coding genes are divergently transcribed with their transcription start sites (TSSs) at a distance <1 kb [1–3]. “Head-to-head” (H2H) is used to describe a gene configuration where two adjacent genes are located on opposite strands of DNA and transcribed divergently with TSSs <1 kb. The sequences between an H2H gene pair (intra-H2H pair) are called bidirectional promoters. The region spanning both genes and the bidirectional promoter region is regarded as an H2H block; genes with H2H organization are called H2H genes (see Figure 1A). Two distinct H2H gene pairs comprise an inter-H2H pair (see Figure 1B). Our previous work has reported that H2H gene pairs are evolutionally conserved, functionally related and co-expressed [2, 3]. The mechanism of transcriptional regulation of intra- and inter-H2H gene pairs is still poorly understood and deserves considerable attention.

It is thought that intra-H2H gene pairs are regulated by bidirectional promoters [4]. But what elements and transcription factors (TFs) play a vital role during the regulation process is still an open question. It is well known that eukaryotic gene expression regulation involves combinatorial control of TFs, which could be organized both in linear and three-dimensional conformations [5]. In our previous report, there proved to be positive expression correlations among H2H gene pairs as well as genes within an individual H2H pair [3]. However, the means by which TF regulations can be accomplished over long distances between inter-gene pairs are unknown. It was hypothesized that the establishment of close contacts or chromatin loops may facilitate the process [6]. Using the new Hi-C technology, spatial proximity maps of the human genome have been built [7]. With ChIP-seq data, TFs and modified histones for H2H genes can be identified. By using these data, we identified two distinctive groups of transcription factors mediating intra- and inter-H2H gene transcriptions respectively.

It has been proposed that bidirectional promoters should not be considered under an umbrella classification for one large regulatory network, nor should they be divided into thousands of gene pairs [8]. We hypothesized that such H2H genes, may contribute to the compactness of the overall gene regulatory network because they are highly co-expressed. Through the construction of human co-expression networks as part of our methodology, we looked into the connections between H2H genes and non-H2H genes and compared their attributes.

## Results and discussion

### Characterization of TFs for intra-H2H gene pairs

**Figure 2:**
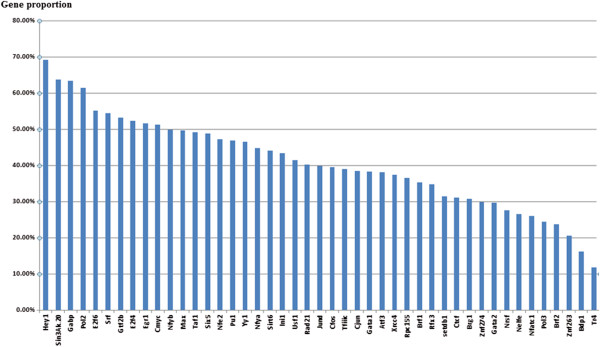
**The distribution of H2H gene pairs by transcription factors bound to H2H gene pair blocks.**

In this study, there were 1447 Human H2H pairs in DBH2H in total [9] and 45 transcription factors are available by using the public ChIP-seq data in the K562 cell line (see Methods). If the binding sites of one TF overlap with H2H blocks, the TF is regarded to bind to these H2H gene pairs. Out of the 1447 H2H pairs, a total of 1308 were identified to bind to the 45 TFs. We defined TFs that bind to more than 45% of H2H pairs as overrepresented TFs, of which there were 17 overrepresented TFs identified (see Figure 2). Overrepresented transcription factors such as GABP, POL2, E2F6, E2F4 and c-MYC have been reported in previous studies [4], and transcription factors like HEY1, SIN3AK-20 and SRF were first discovered in our study. As we know, H2H genes are mainly enriched in biological processes like DNA repair and the cell cycle (see the function enrichment results using DAVID [10] in Additional file 1). Overrepresented TFs are mainly involved in active transcriptional activity or regulating genes in the cell cycle process. GABP is the ubiquitous ets-family transcription factor GA-binding protein. It was reported that GABP binding affinity is associated with bidirectional transcriptional activity in a luciferase transfection assay [11]. POL2 is RNA polymerase II which catalyzes the transcription of DNA to synthesize precursors of mRNA [12]. c-MYC target genes are mainly involved in cell growth, apoptosis and metabolism [13]. The E2F family plays a crucial role in the control of the cell cycle and tumor suppressor protein activities [14]. E2F4 and E2F6 are involved in the cell cycle regulation. According to Gene Ontology (http://www.geneontology.org/), protein HEY1 is an RNA polymerase II core promoter sequence-specific DNA binding transcription factor. SRF is a transcription factor that binds to the serum response element (SRE). This protein regulates the activity of many immediate-early genes, and participates in cell cycle regulation, apoptosis, cell growth, and cell differentiation [15].

**Figure 3:**
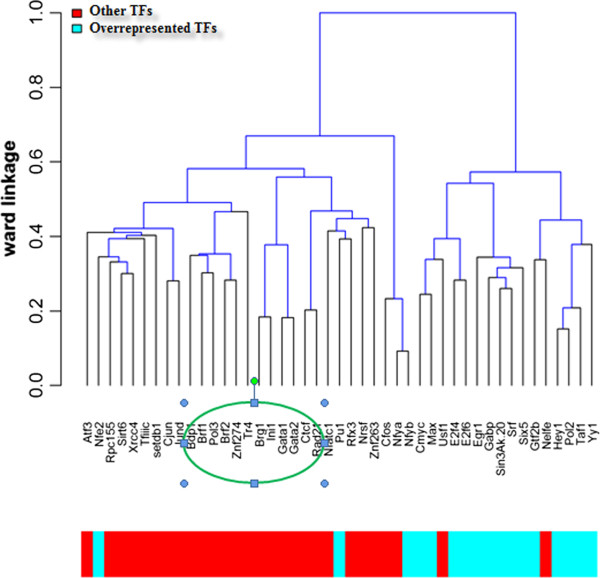
**Hierarchical Clustering of TFs bound to H2H gene pairs.** Overrepresented TFs are TFs that bind to more than 45% of H2H pairs while other TFs are the non-overrepresented TFs.TFs in the green ellipse are TFs preferring to interact with interacting inter-H2H pairs versus intra-H2H pairs and they all belong to the ‘other TFs’ group.

Based on the regulation profile of TFs binding to H2H gene pairs, we clustered the TF pairs hierarchically using Pearson’s correlation coefficient and Ward’s method [16]. As shown in Figure 3, 45 TFs were clustered into 2 groups, one group mostly comprised of overrepresented TFs (coloured in Blue) and the other group mostly comprised of non-overrepresented TFs (coloured in Red).

### TF interaction network regulating H2H gene pairs

Using the transcription factor data described in Methods, we built a binary matrix categorizing whether a given H2H gene pair is regulated by a given TF. By analyzing the correlation between each TF pair (see Methods), we identified co-occurring TF pairs which bind to the same H2H gene pairs. Then we constructed a transcription factor interaction network based on TFs’ co-occurrence.

**Figure 4:**
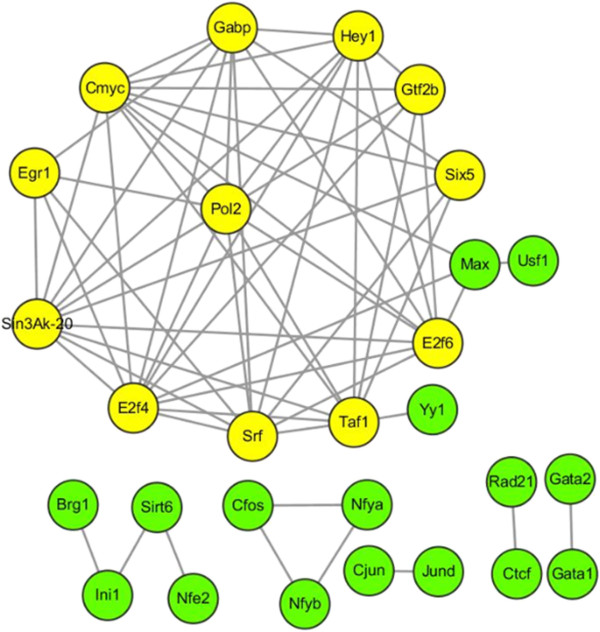
**A co-functional network of H2H gene pair transcription factors.** In the network, yellow nodes represent TFs with high degree and green nodes refer to TFs with small degree.

As shown in Figure 4, 12 TFs (nodes in yellow) are highly connected to each other, and each of them regulates more than 40% of all H2H genes. All these TFs co-regulate H2H genes and most of them are essential for bidirectional promoters in the regulation of intra- H2H genes. TFs in other small subnets are not included in the overrepresented group. However, based on the evidence in the existing literature, most of these TFs are heavily involved in spatial interactions. One subnet is composed of two TFs including CTCF-RAD21 and another subnet is made up of GATA1-GATA2. Recent genome-wide assays have shown that the transcription factor CTCF can link chromatin domains through long-range interactions between distal genomic regions, suggesting its crucial role as an architect of chromatin conformation. CTCF/RAD21 mediated insulators maintain the chromatin loop formation and the localization of transcriptional apparatuses at the promoters, suggesting the essential role of chromatin insulation in controlling the expression of clustered genes [17]. GATA was demonstrated to regulate genes remotely. The knockdown of GATA1 and GATA2 altered the expressions of genes close to GATA linearly and spatially [18]. BRG1, connected with other TFs in the subnet (BRG1-Ini1-Sirt6-Nfe2), is a protein required for the long-range interaction of a locus control region with a downstream promoter [19]. TFs Cjun and JunD are connected in the TF interaction network. Cjun, in combination with c-Fos, forms the AP-1 early response transcription factor; and JunD is a functional component of the AP1 transcription factor complex [20]. It is reported that long-range transcription controls of Ap-1 factors play a role in the regulation of the gene PAD/3 [21].

Since TFs can bridge both promoters and distal cis-regulatory elements such as enhancers, insulators, and silencers while looping out of the intervening DNA [17], we looked into the TF distributions in spatially interacting inter-H2H pairs.

### Inter-H2H gene pairs involved in Hi-C supported interaction loci

**Figure 5:**
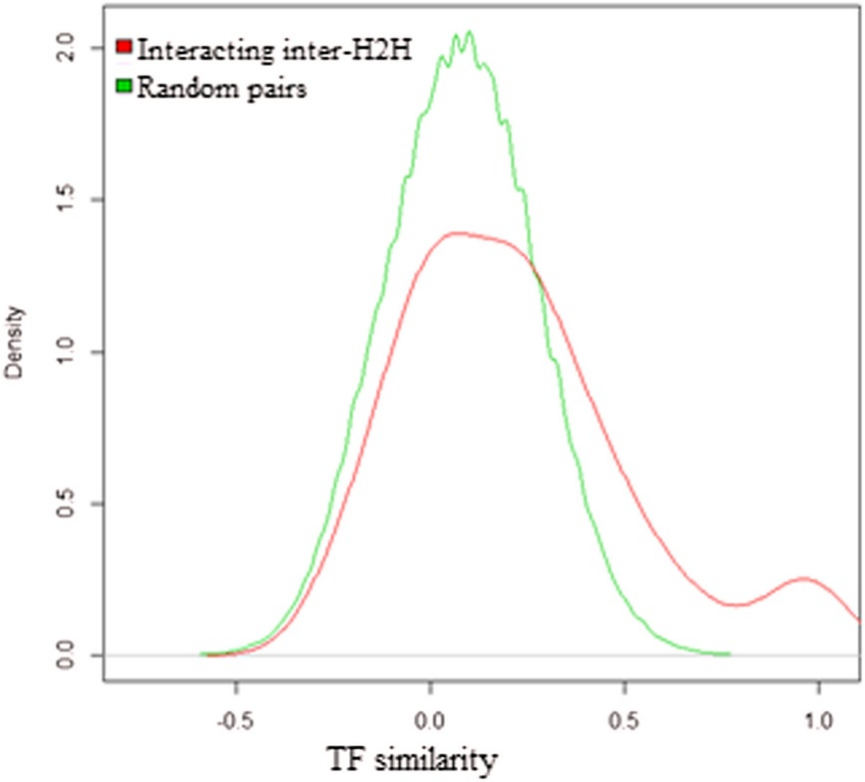
**TF similarity distribution of interacting inter-H2H pairs and random inter-gene pairs.**

Using interacting genomic regions from Hi-C data [18], we mapped H2H gene blocks to long-range interacting loci and identified inter-H2H genes involved in spatial interactions (see Methods). If both of the interacting loci overlapped with different H2H blocks, the two H2H pairs (an ‘inter-H2H’pair) were annotated to be spatially interacting with each other. In this way, a total of 105 interacting ‘inter-H2H’ pairs with Hi-C evidence were selected (see Additional file 2). To identify the characteristics of those inter-H2H pairs, we compared the TF similarities between interacting inter-H2H pairs and random inter-pairs. A “random inter-pair” are two human genes randomly picked out from all the human genes. The interacting inter-pairs had higher TF similarity scores (see Methods) than random inter-pairs (see Figure 5). The average TF similarity score for interacting inter-pairs is 0.221 while it is 0.079 for random inter-pairs. The result indicates that the spatial interaction of inter-H2H genes may be associated with the shared transcription factors.

As shown in Table 1, TFs are ranked by fold changes calculated according to the proportions of TFs binding to inter-H2H pairs compared with TFs binding to intra-H2H pairs. The top TFs with a fold change >2 are BDP1, CTCF, GATA2, BRF1, ZNF274, BRF2. It seems that these TFs may have a stronger preference for binding to inter-H2H vs. intra-H2H. They were clustered in the group marked Red in Figure 3 and differed from the group of overrepresented TFs for bidirectional promoters marked Blue. Some of these TFs in the Red group are indicated in the literature to mediate spatial interactions. CTCF mediates both intra- and inter-chromosomal interactions [22]. ZNF274 is involved in the recruitment of KAP1 and SETDB1 to specific regions [23]. BRF2, encoding one of the subunits of RNA polymerase III transcription factor complex, is a general activator of RNA polymerase III transcription [24]; and many POL3-transcribed genes are found spatially clustered at or near the nucleolus [25]. We infer that other TFs enriched in interacting inter-H2H pairs and clustered with those reported TFs (see green ellipse in Figure 3) may also mediate spatial interactions.

The Hi-C results indicate that intra- and inter-H2H pairs show different transcriptional regulation profiles. The TFs of H2H genes can be separated into 2 groups. One group (marked Blue in Figure 3) mainly regulates linear intra-H2H gene pairs and the other (marked Red in Figure 3) is involved in the spatial regulation of inter-H2H pairs. Transcription factors such as GABP, POL2, SRF, SIN2AK-20, HEY1 and c-MYC regulate bidirectional transcription and are enriched in intra-H2H gene pairs, while other factors such as CTCF, BDP1 and ZNF274 play important roles in spatial interactions between H2H pairs.

**Table 1:** **Percentage of TFs detected in intra- H2H gene pairs and interacting inter- H2H pairs**

TFs	Intra- H2H pairs	Interacting inter-H2H pairs	Enrichment (fold change)
**Bdp1**	14.7%	40%	2.72
**Ctcf**	28.2%	60%	2.13
**Brf2**	21.5%	45.7%	2.12
**Nfatc1**	23.6%	49.5%	2.10
**Brg1**	27.9%	58.1%	2.08
**Znf274**	27.0%	56.2%	2.08
**Gata2**	26.9%	55.2%	2.05
**Tr4**	10.7%	21.9%	2.04
**Znf263**	18.6%	38.1%	2.04
**Nrsf**	25%	50.5%	2.02
**Cjun**	34.8%	69.5%	2.00
**Pol3**	22.1%	42.9%	1.94
**Rfx3**	31.5%	60%	1.90
**Rpc155**	33%	62.9%	1.90
**Jund**	36.1%	68.6%	1.90
**Gata1**	34.6%	64.8%	1.87
**Nelfe**	24%	44.8%	1.87
**Rad21**	36.5%	67.6%	1.85
**Atf3**	34.5%	63.8%	1.85
**Ini1**	39.2%	72.4%	1.84
**Yy1**	42.1%	77.1%	1.83
**Max**	45.1%	82%	1.82
**Cfos**	35.8%	63.8%	1.78
**Pu1**	42.5%	75.2%	1.77
**Tfiiic**	35.2%	61.9%	1.76
**Sirt6**	39.9%	69.5%	1.74
**Brf1**	31.9%	54.3%	1.70
**Nfya**	40.6%	68.6%	1.69
**Nfyb**	45.1%	76.2%	1.69
**Xrcc4**	33.9%	57.1%	1.69
**Six5**	44.2%	74.3%	1.68
**Taf1**	44.5%	74.3%	1.67
**E2f4**	47.4%	78.1%	1.65
**Setdb1**	28.5%	46.7%	1.64
**Nfe2**	42.8%	69.5%	1.63
**Cmyc**	46.4%	74.3%	1.60
**Usf1**	37.6%	60%	1.60
**Egr1**	46.7%	74.3%	1.59
**Srf**	49.3%	78.1%	1.58
**Pol2**	55.6%	86.7%	1.56
**E2f6**	49.9%	76.2%	1.53
**Hey1**	62.6%	92.4%	1.48
**Gtf2b**	48.2%	70.5%	1.46
**Gabp**	57.4%	82.9%	1.44
**Sin3Ak-20**	57.6%	82.9%	1.44

The proportion of intra- or inter- pairs bound by one TF is the number of intra-/inter- pairs bound by one TF divided by the total number of intra- or inter- pairs.

### Modified histones and hypersensitive DNase bound to H2H gene pairs

1384 of the 1447 H2H pairs are associated with the 9 histones and DNase. The distribution of histones and DNase bound to H2H pairs is described in Table 2. Hypersensitive DNase, H3K4me2, H3K4me3, H3K9ac and H3K27ac, which are related to transcriptional activation, bind to > =80% H2H pairs; while H3K27me3 and H3K9me3, which are associated with gene silencing [26], are only bound to a small fraction of H2H gene pairs. Compared to the background genes, a higher proportion of H2H gene pairs bind to a number of epigenetic markers, such as hypersensitive DNase, H3K4me2, H3K4me3, H3K9ac and H3K27ac (see Table 2, p < 0.01 using Chi-square test). Previous studies have reported that H3K4me3 islands overlap with about 85% of active genes (expressed genes) while overlapping with 59% of silent genes (non-expressed genes); H3K27me3 and H3K9me3 protein levels were much higher at silent promoters than at active promoters [27]. The above data suggest that H2H gene pairs, bound by modified histones of transactional activation, should be highly expressed. An analysis of whole genome microarray data validated that 68% of H2H genes are transcribed compared to only 44% of all human genes [4].

**Table 2:** **The proportion of hypersensitive DNase and modified histones bound to H2H pairs**

Epigenetic markers	#H2H pairs with epigenetic markers	Proportion of H2H with epigenetic markers	Proportion of all genes with epigenetic markers
**H3K4me2**	1221	84.38%	48.92%
**H3K4me3**	1218	84.17%	47.86%
**H3K9ac**	1174	81.13%	43.47%
**H3K27ac**	1148	79.34%	42.30%
**H3K4me1**	986	68.14%	41.39%
**H3K36me3**	784	54.18%	29.37%
**H4K20me1**	687	47.48%	31.06%
**H3K27me3**	199	13.75%	17.13%
**H3K9me3**	63	4.35%	4.84%

### The role of H2H genes in Human co-expression network

**Figure 6:**
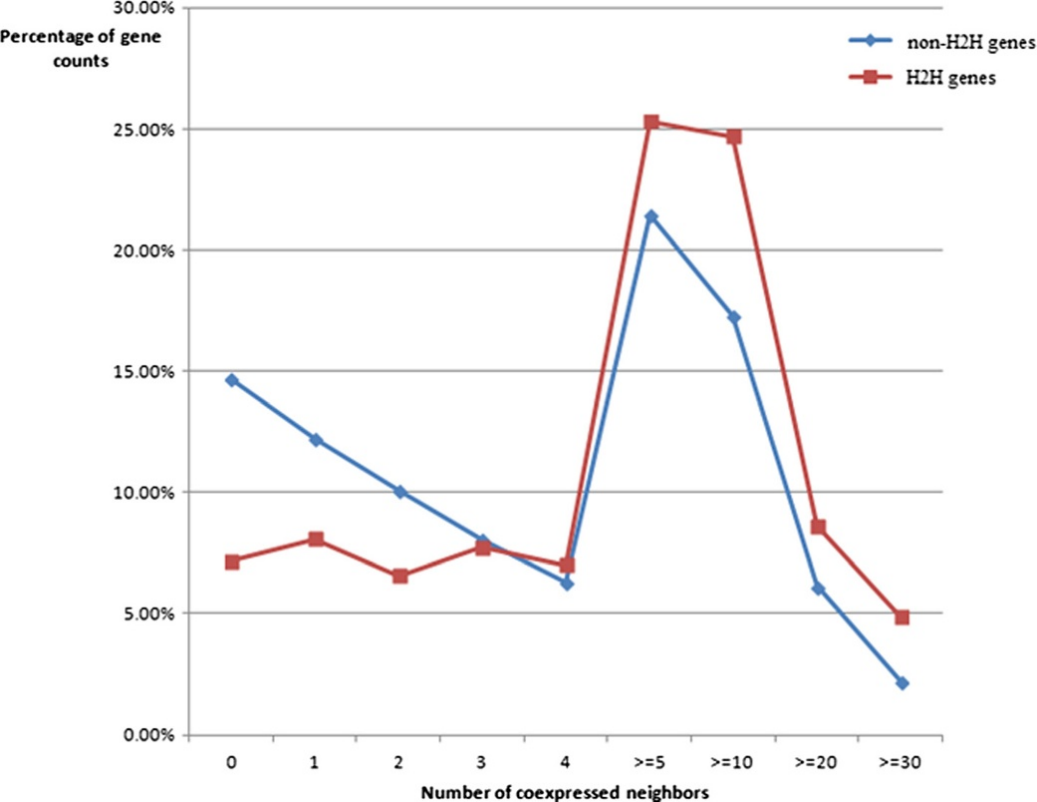
**Percentage of gene counts by number of co-expressed neighbors.** Non-H2H genes are genes without H2H gene organization.

Since inter-H2H gene pairs interact spatially and H2H gene pairs are biologically important, we speculate as to whether H2H genes are also highly connected to other genes. In this work, we constructed a human co-expression network of 20280 genes (see Methods) and calculated the number of co-expressed neighbours of each gene. The percentage of gene counts by number of co-expressed neighbours is shown in Figure 6. As it makes clear, H2H genes exhibited higher connectivity than non-H2H genes. The average numbers of co-expressed neighbours for H2H genes and non-H2H genes are 7 vs. 4 (median) or 9.8 vs. 7.6 (mean). The Wilcoxon signed-rank test showed the difference between the two groups (H2H genes vs. non-H2H genes) was statistically significant (p-value < 2.2e-16). We also randomly selected genes equal to the number of H2H genes from all genes in the network to compare the amount of co-expressed neighbors between the two groups, repeating this function 10000 times. H2H genes still showed a higher average connectivity each time (see Additional file 3). From the above result, we conclude that it is likely that H2H genes are highly connected with other genes (not only H2H genes) in a human co-expression network.

It was reported that co-expressed genes tend to be co-regulated by one or more common transcription factors [28]. Since H2H genes tend to be highly connected to other genes in the co-expression network and have distinctive groups of transcription factors mediating intra- and inter-H2H gene transcriptions, we propose that H2H genes contribute to the compactness of the overall gene regulatory network.

## Conclusions

A systematic investigation of H2H genes, their transcription factors and the histones and DNase bound to them based on human genome Hi-C, ChIP-seq and DNase-seq data was conducted in this study. We echoed and adjusted several known properties of H2H gene organization and also provided new observations on the spatial regulation of H2H genes. We further demonstrated that H2H intra-pairs and inter-pairs are regulated by two distinct groups of transcription factors. The binding of hypersensitive DNase and the modified histones associated with active transcription may facilitate the high expression of H2H genes. Finally, we analysed the properties of H2H genes in a human co-expression network and found that H2H genes tend to be highly connected to other genes. We propose that the highly expressed H2H genes, regulated through both linear and spatial interactions, contribute to the compactness and thus the high efficiency of the entire gene regulatory network.

## Methods

### Data sources

H2H gene pair information was obtained from our previous work: DBH2H [9]. DBH2H contains information about H2H gene pairs from species Human, Mouse, Rat, Chicken and Fugu. There are 1447 H2H pairs in the DBH2H database. Human gene co-expression data was obtained from COXPRESdb [29]. COXPRESdb is a database of co-expressed gene sets. Gene expression profiles of humans in the database are from NCBI GEO, based on the Affymetrix GeneChip (Human Genome U133 Plus 2.0 Array). Genomic interaction regions calculated by Xun Lan et al. were derived from an Hi-C data set in K562 cell line [7] using the Mixture Poisson Regression Model and a power-law decay distribution [18].

DNase-seq data for DNase hypersensitivity and ChIP-seq data for 9 modified histones and 45 transcription factors in the K562 cell line were downloaded from UCSC (http://genome.ucsc.edu/encode/dataMatrix/encodeDataMatrixHuman.html). The data were analyzed using W-ChIPeaks (http://motif.bmi.ohio-state.edu/W-ChIPeaks) [18, 30].

### Identification of interacting inter-H2H pairs

We integrated the location data of human H2H blocks with genomic interaction loci from Hi-C. If a locus overlapped with a H2H block, we annotated the locus with the H2H pair. If both of the interacting loci overlapped with different H2H blocks, the two H2H pairs (an ‘inter-H2H’pair) were regarded to be spatially interacting with each other. A total of 546 pairs of interacting loci were fully annotated by H2H pairs, and among them, 105 pairs of interacting loci were annotated by different H2H pairs (‘inter-H2H’ pairs) (see Additional file 2).

### Histone modifications and transcription factors for H2H genes

In this paper we studied 45 transcription factors, 9 different types of histone modifications as well as DNase hypersensitive sites. TFs, modified histones and DNase with binding sites overlapping with H2H blocks were identified and annotated with H2H pairs for further analysis. For an interacting ‘inter-H2H’ pair, TFs bound to either H2H pair were regarded as binding to the ‘inter-H2H’ pair. The distributions as well as the preferences of transcriptional factors and epigenetic markers bound to H2H pairs were investigated. To assess the bindings of epigenetic markers to background genes (all the 25363 human genes from UCSC hg18), we used each human gene region plus its 100 bp bases before transcription start site to map to the binding sites of modified histones and DNase. We also compared TF similarities between interacting inter-H2H pairs and random inter-pairs to study the characteristics of their spatial interactions. Here, the TF similarity between an inter-gene pair is represented as the ratio of the shared TFs to the total TFs binding to the same inter-gene pair.

### Construction of co-functional networks by transcription factors

We obtained a binary regulation matrix of transcription factors and H2H genes after completing the above analysis (see Additional file 4). The matrix rows represent transcription factors and the columns represent H2H gene pairs. Each element of the matrix indicates the binding of a transcription factor to an H2H gene pair. 1 represents a transcription factor binding to an H2H pair, 0 represents a transcription factor not binding to an H2H pair. Then we analyzed the co-occurrence of each transcription factor pair regulating at least 10 gene pairs. P-value was calculated to evaluate the statistical significance of co-occurrence using a binomial test. The overlapping ratio between a pair of two transcription factors was calculated as: number of shared H2H pairs regulated by a TF pair/total number of H2H pairs bound to a TF pair. We defined transcription factor pair co-occurrence as a calculated p-value < 0.01 and the overlapping ratio > 0.6. A co-occurrence network of transcription factors that regulate H2H genes was visualized using Cytoscape 3.0.2 [31].

### Human co-expression network

COXPRESdb provides the co-expression data of 20280 human genes including Pearson’s correlation coefficient (PCC) of gene expression profiles and a relative correlation index: mutual rank (MR) for each gene [32]. Mutual rank (MR) is a geometric average of the PCC rank from gene A to gene B and that of gene B to gene A and is considered a standard measure of the biological significance of gene co-expression. Here, we considered gene pairs with MR < =20 as co-expressed pairs. We constructed a global human gene co-expression network based on the co-expressed pairs and singleton genes (without co-expressed genes) from this database.

## Electronic supplementary material

Additional file 1: **Functional annotation for H2H genes.** The function enrichment results for H2H genes using DAVID are listed in the table. (XLS 182 KB)

Additional file 2: **Inter-H2H pairs with spatial interaction.** Using interacting genomic regions from Hi-C data, 105 inter-H2H pairs with spatial interactions were identified (see the first sheet). And the detailed H2H pair information is also provided on another sheet. (XLS 370 KB)

Additional file 3: **The distribution of the average number of co-expressed neighbours of the randomized genes in the co-expression network.** The red line represents the mean number of co-expressed neighbors of H2H genes and the black curve is the distribution of the average number of co-expressed neighbors of randomized genes. (PNG 29 KB)

Additional file 4: **A binary matrix of TFs regulating H2H gene.** A binary regulation matrix of transcription factors (TFs) and H2H genes is provided in sheet 1. Row names are H2H gene pairs, column names are TFs. The value of the matrix indicates whether a transcription factor is binding to an H2H gene pair. 1 represents a transcription factor binding to an H2H pair, 0 represents a transcription factor not binding to an H2H pair. (XLS 467 KB)

## Abbreviations

TF: Transcriptional factor
H2H: Head-to-head
PCC: Pearson’s correlation coefficient
MR: Mutual rank.
