# Supplementary figures and images for: Transcriptional regulation and spatial interactions of head-to-head genes
Source: BMC Genomics. 2014 Jun 24;15(1):519. doi: 10.1186/1471-2164-15-519 (PMC4089025; doi:10.1186/1471-2164-15-519)

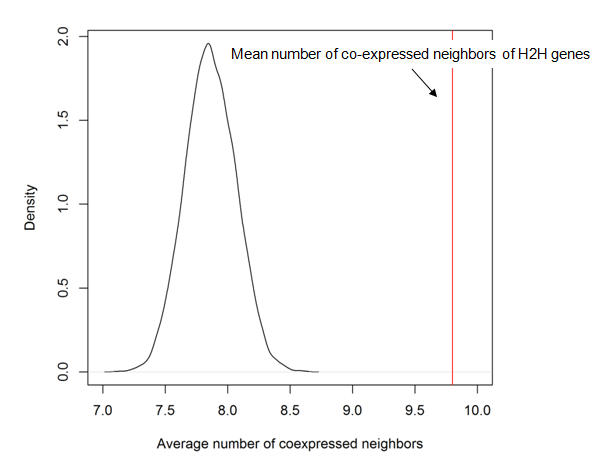

Supplement: Supplementary file 3 — Additional file 3: The distribution of the average number of co-expressed neighbours of the randomized genes in the co-expression network. The red line represents the mean number of co-expressed neighbors of H2H genes and the black curve is the distribution of the average number of co-expressed neighbors of randomized genes. (PNG 29 KB) [file 12864_2014_6207_MOESM3_ESM.png]
